# Complications of Single-Use Flexible Ureteroscopy vs. Reusable Flexible Ureteroscopy: A Narrative Review

**DOI:** 10.7759/cureus.76256

**Published:** 2024-12-23

**Authors:** Ana Maria Punga, Cosmin Ene, Catalin - Andrei Bulai, Dragos A Georgescu, Razvan Multescu, Dragos Eugen Georgescu, Bogdan Geavlete, Petrisor Geavlete

**Affiliations:** 1 Department of Urology, Carol Davila University of Medicine and Pharmacy, Bucharest, ROU; 2 Department of Urology, "Sf. Ioan" Clinical Emergency Hospital, Bucharest, ROU; 3 Department of General Surgery, Carol Davila University of Medicine and Pharmacy, Bucharest, ROU; 4 Department of General Surgery, "Dr. I. Cantacuzino" Clinical Hospital, Bucharest, ROU

**Keywords:** flexible ureteroscopy, procedural complications, reusable, single-use flexible ureteroscope, urolithiasis

## Abstract

Urolithiasis, or kidney stones, is a painful condition that is becoming increasingly common worldwide. For many, the solution lies in a minimally invasive procedure called flexible ureteroscopy (fURS). This technique involves inserting a tiny, flexible scope into the urinary tract to break up and remove stones. Reusable fURS scopes have traditionally been the norm. However, concerns about infection control and instrument durability have led to the development of single-use scopes. While both methods offer effective treatment, the question remains: which one is safer and more efficient? To answer this, we conducted a comprehensive review of the available research. We analyzed 37 studies that compared single-use and reusable fURS complication rates. While both methods carry risks, such as bleeding, infection, and ureteral injury, the overall complication rates were found to be similar. As technology continues to advance, fURS is becoming even safer and more effective. However, there is still a need for standardized reporting and further research to better understand the potential risks and benefits of both single-use and reusable scopes. Ultimately, the choice between the two will depend on various factors, including patient factors, surgeon preference, and healthcare resource availability.

## Introduction and background

Urolithiasis represents a health problem that has recently continued to rise globally [1]. Men are more likely to develop kidney stones than women are (10.6% vs. 7.1%). The prevalence increases with age [2].

For stones smaller than 1 cm or between 1 and 2 cm in diameter, commonly used treatments include ureteroscopic fragmentation or shockwave lithotripsy. Ureteroscopy generally has a higher success rate in achieving a stone-free state compared to shockwave lithotripsy [3,4]. For treating renal calculi greater than 2 cm in diameter percutaneous nephrolithotomy (PCNL) is recommended [5].

Flexible ureteroscopy (fURS) has become a preferred treatment option for renal stones over PCNL, primarily due to the higher complication rate associated with PCNL, including potentially life-threatening complications like urothorax and bleeding [6]. Reusable digital flexible ureteroscopes have been adopted as a method of urolithiasis treatment by an increased number of urologists [7].

A major issue with using reusable flexible ureteroscopes is represented by the sterilization procedure [8]. Contamination (by bacteria, hemoglobin, adenosine triphosphate, and protein) could still be identified in the tested reusable ureteroscopes sometimes even after they were cleaned manually and sterilized using hydrogen peroxide gas [9]. In this context, a single-use flexible ureteroscope became the solution to this problem.

Recently, single-use fURS were developed as an alternative to reusable ureteroscopes to remove the risk of cross-infection [10]. Single-use fURS are intended to reduce the drawbacks of reusable fURS, their high chance of breaking, and the expenses associated with purchase and maintenance [11].

In the routine practice of daily endourology, fURS proves to be a reliable and secure method for treating upper urinary tract stones. Nonetheless, it is essential to acknowledge that the likelihood of complications rises in cases of substantial stone burden or multiple stones. Furthermore, caution should be exercised to avoid overstating the incidence of complications associated with this procedure. The review aims to evaluate the existing experience from the literature to find out as much as possible about existing complications for single-use fURS in comparison with multiuse fURS [12].

## Review

Material and methods

Using the Pubmed and Embase databases, a thorough literature search was conducted to find original, peer-reviewed studies that examined the effectiveness of single-use flexible urinareoscopy for the assessment and management of urinary tract calculi. The following search algorithm was used: “single use,” “flexible scope,” “ureteroscopy,” “pyeloscopy,” or “ureteropyeloscopy.” The full texts of possibly qualifying studies were collected after two writers separately reviewed the citations' titles and abstracts. Differences were settled through discussion. The most current publication was selected when a patient group was mentioned twice. The corresponding author was contacted to provide clarification on any unclear or incomplete data. To locate pertinent papers, the PubMed literature database was searched. To identify related publications concerning the complication of reusable and single-use flexible ureteroscope treatment, the search terms “single-use flexible ureteroscopy complications” OR “reusable flexible ureteroscopy complications” OR “single-use vs. reusable ureteroscopy” were included to attain relevant studies. To evaluate and compare mechanical, optical, and clinical outcomes, comparative studies were also included between traditional (i.e., non-disposable) flexible ureteroscopes and single-use flexible ureteroscopes. Other inclusion criteria used to choose the final studies were language limited to English; renal stones treated using reusable and/or single-use fURS and laser lithotripsy, there were no restrictions on the number or location of the stones or the type of scopes used. In this paper, there were no included studies presented as conference presentations or abstracts, or any studies with neoplasia patients. Thirty-five researchers were eliminated after the initial evaluation of 72 papers. As a result, our final analysis comprised 37 studies.

Results

Use of Ureteral Access Sheaths (UAS)

Zhu et al. outlined a sheath incorporating both suction and pressure venting, with an inner obturator size of 12F and an outer sheath size of 14F. They performed a matched-pair analysis on 165 patients undergoing reusable fURS (Olympus, Tokyo, Japan), comparing outcomes between a suctioning ureteroscope and a standard ureteroscope. The suction UAS group exhibited a lower overall complication rate (11.5% vs. 24.8%) and fewer postoperative infectious issues [13]. The theory of irrigation and suctioning suggests that utilizing suctioning ureteral access sheats may contribute to lowering intrarenal pressure and enhancing surgical visualization [14]. Expanding the diameter of the access sheath does not enhance intrarenal irrigant flow when the flexible ureteroscope's working channel accommodates a basket. Somani et al. have introduced two configurations designed to improve irrigant flow with a larger basket in place while keeping intrarenal pressures low, recommending using a 10F peel-away sheath in combination with a 4F ureteral access catheter for irrigation inflow placed alongside, which is suitable for nearly all cases.

Employing lower operating pressures reduces the risk of pyelo-venous backflow and minimizes damage to the collecting system. This, in turn, decreases the likelihood of systemic inflammatory response and sepsis, attributed to the reduced spread of bacteria [15].

Intrarenal Pressure

Excessive irrigation or pressure during the procedure can lead to over-inflation of the renal pelvis, potentially causing increased intrarenal pressure. This may result in complications such as renal pelvis rupture or extravasation of contrast material.

The potential risk of perioperative sepsis arises from the introduction of bacteria and endotoxins into the bloodstream, especially in the presence of elevated intrarenal pressure [16]. Reducing perfusion flow to mitigate high intrarenal pressure can compromise surgical visualization and lead to decreased lithotripsy efficacy. The conventional endorsement of UAS in fURS is based on its role in facilitating the procedure and reducing intrarenal pressure. However, there is limited evidence regarding the impact of UAS on perioperative infective complications and stone-free rates (SFR) [17]. Complications are intricately linked to the interplay between the distinctive features of normal upper urinary tract physiology and the specific elements inherent in fURS, namely access and irrigation. Understanding this interrelation is essential for preventing or mitigating potential adverse effects associated with fURS [18].

Instrument Malfunction

The flexible ureteroscope and associated instruments are delicate and can be prone to technical issues such as malfunction, breakage, or damage during the procedure. This can interrupt the surgery and may necessitate additional steps to address the problem.

Inadequate visualization: Limited visibility within the urinary tract due to bleeding, stone debris, or other factors can pose challenges during the procedure. Clear visualization is crucial for the accurate identification and treatment of stones or other abnormalities.

In an 11-year study, Juliebø-Jones et al. identified 206 events related to flexible ureteroscopes (147 reusable, 56 single-use) from 14 manufacturers. Patient injuries occurred in 21.8% of cases, exclusively involving reusable scopes (avulsion, ureteral perforation, bleeding, septic shock, scope entrapment). The most common issue, observed in 26.7% of cases, was a complete loss of image. Other frequent challenges included scope removal difficulties (13.6%), scope damage from the basket (4.4%), distal tip detachment (5.8%), contamination (4.9%), and deflection failure (4.9%). Surgeon error (45.6%) was the primary cause of these events, surpassing device malfunction (26.2%). Single-use scopes were more likely to experience issues related to manufacturing defects, such as complete image loss and power outages [19].

The study suggests that the most common scope failure encountered was the absence of image display when the scope was connected (three instances). Additionally, damage to the deflection mechanisms of the flexible ureteroscope was reported in three cases. Interestingly, the study found no significant discrepancy in these issues between single-use and reusable scopes. This implies that both types of scopes were susceptible to similar problems in the described scenarios. Some of the mechanical complications were represented by the deflection handle breaking compromising the deflection mechanism, the scope shaft breaking, unable to navigate extremes of the pelvic kidney, no image when the scope plugged into the tower, laser breaking in the channel and lasered through the scope [20].

Ureteral Injury or Perforation

The flexible ureteroscope may inadvertently cause injury or perforation of the ureter or adjacent structures. This risk is higher when navigating through tight or tortuous passages. Among the 1,571 procedures, Bas et al. observed 209 cases of complications, constituting 13.3%. The overall rate of intraoperative complications stood at 5.9%. Notably, the primary complications encountered were bleeding (2.5%) and mucosal injury (2.3%). Perforation, necessitating JJ stent insertion, and instrument malfunction or breakage were identified at a minimal incidence of 0.3% and 0.1%, respectively [12]. In another study, conducted by Giulioni et al., there were 73 (2.5%) ureteral injuries requiring prolonged stenting out of the 2,946 patients treated with reusable scopes [21].

Despite being described as uncommon complications in the literature, ureteral perforations and fornix ruptures were reported in a 2020 study by Huang et al., who performed 363 flexible ureteroscopies on 271 patients. These authors used the 9.9F flexible ureteroscope (Olympus) through a 13/15F ureteral access sheath [22].

Hematuria

Hematuria, or blood in the urine, is a common immediate postoperative complication. In two cases from Huang's study involving reusable fURS for 2 cm calculi, the ureteral perforations were self-limiting [22]. Kam et al. found no significant difference in the rate of procedures terminated due to bleeding or loss of vision between reusable and single-use flexible ureteroscopes [20].

Ureteral Edema

Irritation and manipulation of the ureter during the procedure may lead to edema (swelling) in the ureter. This can contribute to postoperative pain and may affect urine drainage. Patients often experience varying degrees of pain or discomfort after fURS. Adequate pain management, including analgesic medications, is essential to ensure patient comfort.

Bosquet et al. noted two renal colics requiring immediate hospitalization for analgesic medical treatment [23]. Baboudjian et al. noted a small difference in the appearance of flank pain after a single use and reusable ureteroscopy 10 vs five patients [24].

Acute Retention of the Urine

Bosquet et al. described acute retention of urine requiring hospitalization for bladder catheterization after reusable ureteroscopy was performed in an ambulatory regimen [23]. 

Urinary Tract Infection (UTI)

Infectious complications, instances of fever, and urinary infections (Clavien grade 2), necessitating extended antibiotic therapy, were observed in 169 patients (5.7%). Conversely, sepsis leading to intensive-care admission (Clavien 4) was identified in 33 patients out of 2,946 (1.1%) reusable scopes [21]. The conclusion of a 2017 paper underscores the importance of regular audits of reprocessing procedures conducted by infection prevention and sterile processing experts possessing sufficient expertise to evaluate the effectiveness and timeliness of ureteroscope reprocessing. Technicians should incorporate routine cleaning verification tests and meticulous visual inspections [9].

In a 2013 study, Chang et al. described the isolation of one ertapenem-resistant Enterobacter cloacae from a reusable flexible ureteroscope. Despite revising and implementing disinfection protocols for ureteroscopes, additional UTI cases emerged. The pathogen persisted in subsequent surveillance cultures until the implementation of a revised disinfection protocol, including ethylene oxide sterilization, proved effective in terminating the outbreak [25]. In the case of single-use scopes, this situation can be avoided, or limited because of their particularity, they are sterile from the factory and used only once.

Bosquet noted one case of rehospitalization for acute prostatitis, requiring antibiotic therapy, after reusable fURS with Olympus URF-P5 [23]. Although urosepsis is generally less frequent with single-use ureteroscopes, Baboudjian et al.'s study found a slightly higher incidence in patients using reusable scopes. Cultures identified in these cases included Pseudomonas aeruginosa and Enterococcus spp. (four patients each), and Escherichia coli and Candida spp. (one patient each) [24].

Stricture Formation

Scar tissue formation or strictures in the ureter may occur as a result of the procedure. Strictures can lead to complications such as obstructed urine flow and may require further intervention. Aykanat et al. conducted a prospective comparison of the probability of stricture formation in non-presented patients utilizing a 9.5/11.5 Fr and 12/14 Fr UAS one year later. Even with a 12/14 Fr UAS, which was endoscopically shown to increase the likelihood of high-grade ureteral injuries, ureteral stricture formation did not change after a year of follow-up [26]. Two further retrospective investigations on adults [27] and one in 48 children with a mean follow-up of 17 months [28] reported similar results. In contrast, stricture formation was detected in 3% of patients following a three-month follow-up in a retrospective analysis involving 1,001 retrograde intra-renal surgery (RIRS) [29]. Other than ureteral perforation and surgical duration > 60 min, the use of UAS was determined to be an independent risk factor for stricture formation in multiple regression analysis [29]. Despite the relatively high number of strictures reported in this study compared to previous literature, the authors did not provide any specific comments [30].

Renal Colic

The manipulation of stones or fragments during the procedure may cause temporary obstruction, leading to renal colic. In a study on 13,143 patients who underwent RIRS, factors linked to the prescription of opioids for post-ureteroscopy included the use of a UAS, in addition to age, male sex, greater BMI, lack of a pre-operative ureteral stent, and a stent inserted during surgery [31]. These findings contradict a small prospective study that examined postoperative pain problems following RIRS with 30 participants who used a UAS and 30 participants who did not (n = 21). The study reported no statistically significant difference between the two groups. Oguz et al. [32] reported similar findings. A different study found no correlation between postoperative discomfort and the size of the UAS [33].

Fever

Postoperative fever may indicate an infection or an inflammatory response. Monitoring and investigating the cause of fever is crucial for timely intervention. In the 2020 paper, 9.6% of Huang's patients had a fever that remitted under antibiotics and anti-inflammatory medication [22].

Steinstresse: Although it may occur a rare complication, for instance in a 2016 study nine out of 1,571 cases were described [12]. Jarry et al. describe 58 cases of sepsis. The mean operative time was longer in this group than in the one with no complications. Hospital lengths of stay were longer for patients with a past medical history of UTI [34].

Persistent Hematuria

Prolonged or recurrent hematuria (blood in the urine) may occur after fURS. This may be due to ongoing irritation, persistent stone fragments, or other underlying issues.

In certain instances, hematuria may manifest mildly; however, in more severe cases, it may necessitate blood transfusions, as observed in 181 patients out of the total cohort of 2946 subjects involved in a study utilizing reusable flexible scopes [21]. Persistent hematuria may occur in the use of flexible scopes, such as LithoVue and Eu-scopes, and can be treated conservatively [35].

Stent-Related Complications

If a ureteral stent was placed during the initial procedure, patients may experience late complications such as stent migration, discomfort, or urinary symptoms. These issues may become apparent weeks or even months later. Salvado described one case of double-J stent replacement for pain management in the single-use EU scope group [35]. Stent-related complications cannot be related to the type of ureteroscope used but probably the time of the catheter.

Residual Stones

Despite efforts to remove all stones during the initial fURS, small residual fragments may remain in the urinary tract and contribute to complications such as stone growth or recurrence. In a 2022 study, Salvado et al. concluded that the use of single-use flexible Eu-scopes had the highest SFR from all the single-use scopes used, approximately 81% [35].

Ureteral Avulsion

One of the most difficult is total avulsion of the proximal ureter, which occurs in 0.06-0.45% of patients [36]. Stretching in the ureter's weakest spot, most frequently caused by iatrogenic ureteral injuries, can result in ureteric avulsion [37]. Unfortunately, detailed information regarding the location of the damage, the UAS injury classification based on Traxer and Thomas' prospective study [38], and any unintentional ureteral injuries brought on by the use of various instruments were not disclosed. In this context, it is yet unclear if reusable scopes are associated with a decreased risk of complications. However, the use of particular disposable scopes increased the difficulty of navigating. However, using specific disposable scopes made it more difficult to navigate the pelvicalyceal system, which exacerbated renal mucosal damage [39].

Death

Although mortality after such interventions is rare, it is described in the literature. In 2015, Cindolo et al. conducted a study involving six proficient urologists who documented instances of mortality following fURS in their clinical practice. The most common cause of death was septic shock and respiratory failure. Some of the pathogens described in this study were Proteus mirabilis, Candida glabrata, and multi-resistant E. coli. One 48-year-old male patient died of cardiac arrest after general anesthesia without undergoing RIRS [40]. One death case after reusable fURS was noted, and the cause was septic shock [40].

Discussion

An overall complication rate of 3.5% was reported in a recent study by the Clinical Research Office of the Endourological Society involving 11,885 patients. More precisely, they stated that the rate of Clavien I-II was 2.8%, while the rates of Clavien III and IV were just 0.5% and 0.1%, respectively [41]. When taking the fURS into strict consideration, there is a wealth of information in the recently released literature that describes the use of novel equipment or devices with low and comparable complication rates.

According to Harmon et al. [42], their URS complication rates decreased from 6.6% to 1.5% as a result of the ureteroscope's reduced size. With improvements in ureteroscopic technology, there has been a drop in overall complication rates, with significant complication rates estimated to be between <1% and 1.5%. In addition, it has been reported that the overall PCNL complication rate is as high as 83%, with a serious complication rate of 15% to 20%. For patients whose other modalities have failed, fURS has emerged as the preferred surgery. It is also a practical option for patients who are pregnant, obese, or have anatomic deformities like kyphoscoliosis [43]. Fever (2%-28%), sepsis (3%-5%), steinstrasse (1%), and ureteral injury are the most common complications following ureteroscopy in the peri- and post-operative period [44]. These complications are typically categorized as Clavien-Dindo I and II, or occasionally IIIa when an endoscopic intervention is necessary without general anesthesia [45,46]. Ureteral avulsion, ureteral strictures, kidney injury, fistulas, and severe bleeding with transfusion were among the infrequent consequences (<1%) [47]. The literature provides limited evidence, but there is a growing body of work that describes the clinical experience of major complications in greater detail. This work demonstrates how the presence of major complications was underestimated and is still present in clinical practice. Our group recently published a survey in which they identified and described fatal complications (Clavien V) after URS as extremely rare, but still possible if urologists fail to recognize them in time and limit the potential side effects [40].

Concerning UAS, it is clear that it presents many advantages and major complications related to this, are those that require close monitoring or additional procedures; they are classified as grade II or above of the Clavien-Dindo classification and include perforation, obstructive pyelonephritis, steinstrasse, or subcapsular hematoma. Minor complications are defined as those that would settle on their own or with minimal support or a grade I of the Clavien-Dindo classification, such as self-limiting hematuria or UTI needing antibiotics or analgesics [48]. One limitation of this review is that all included studies were conducted in the past. However, a thorough explanation of the methodology was provided in each study, which may be seen as reducing the possibility of bias. Notwithstanding these drawbacks, the comparison parameters throughout the studies were similar, enabling a meta-analysis of the data to produce a more metaphorical conclusion in addition to a subgroup analysis. An additional constraint pertains to the varied definitions of the SFR across various research. Since all of the trials were from high-volume centers of excellence with skilled endourologists doing the surgeries, it is possible that less experienced centers won't be able to reach such high success rates. Another limitation of this review is the variability in complication rates and evaluation methods across the included studies.

## Conclusions

The fURS has become a widely used and successful procedure worldwide, with thousands of cases performed annually. Nowadays, there is a real debate concerning the advantages and disadvantages of single versus multiuse flexible ureteroscopes. The studied data showed similar complication rates, but further research should evaluate these data using a unified system of criteria.
